# Rotigotine transdermal patch and sleep in Parkinson’s disease: where are we now?

**DOI:** 10.1038/s41531-017-0030-4

**Published:** 2017-09-05

**Authors:** Miguel Rosa-Grilo, Mubasher A. Qamar, Raquel N. Taddei, Javier Pagonabarraga, Jaime Kulisevsky, Anna Sauerbier, K. Ray Chaudhuri

**Affiliations:** 1 National Parkinson Foundation (NPF) International Center of Excellence at King’s College Hospital, London, UK; 2 Department of Basic and Clinical Neuroscience, The Maurice Wohl Clinical Neuroscience Institute, King’s College London and National Institute for Health Research (NIHR) Mental Health Biomedical Research Centre, Institute of Psychology, Psychiatry and Neurosciences, King’s College London, London, UK; 3 Department of Neurology, Movement Disorders Unit, Hospital de la Santa Creu i Sant Pau Sant Pau Institute of Biomedical Research (IIB-Sant Paul). Centro Investigación Biomedica en Red-Enfermedades Neurodegenerativas (CIBERNED), Universitat Oberta de Catalunya (UOC), Barcelona, Spain

## Abstract

A wide range of sleep dysfunction complicates Parkinson’s disease during its course from prodromal to palliative stage. It is now increasingly acknowledged that sleep disturbances are thus integral to the disease and pose a significant burden impacting on quality of life of patients. Sleep fragmentation, restless legs syndrome, nocturia, and nocturnal pain are regarded as one of the main components of night-time sleep dysfunction with possible secondary impact on cognition and well-being. The role of dopaminergic therapies, particularly using a continuous drug delivery strategy in managing some of these sleep issues, have been reported but the overall concept remains unclear. This review provides an overview of several aspects of night-time sleep dysfunction in Parkinson’s disease and describes all available published open-label and blinded studies that investigated the use of rotigotine transdermal patch targeting sleep. Blinded studies have suggested beneficial effects of rotigotine transdermal patch on maintenance insomnia and restless legs syndrome in Parkinson’s disease patients. Open-label studies support these observations and also suggest beneficial effects on nocturia and nocturnal pain.

## Introduction

A range of sleep dysfunctions complicate Parkinson’s disease (PD) and at some stage during the course of the disease, patients may report insomnia, sleep-related breathing disorders, hyper-somnolence, parasomnias, and sleep-related movement disorders.^1^ Rapid eye movement (REM) sleep behavior disorder (RBD), as well as excessive daytime somnolence can be key clinical markers of prodromal PD and be present in untreated early PD together with RLS.^2–4^ Later on, motor symptoms can disturb sleep maintenance. Frequent awakenings at night due to RLS, periodic limb movements in sleep (PLMS) or early morning off (EMO) associated with non-motor symptoms (NMS) likely contribute to the complexity of night-time sleep dysfunction.^5, 6^

Rotigotine (RTG) transdermal patch has been suggested to be efficacious for management of some sleep related problems in PD and is currently the only therapeutic strategy examined with the PD Sleep Scale 2 (PDSS-2) as a primary outcome measure in the RECOVER study, which is a randomized placebo-controlled double-blinded trial.^7^

In this review, we examine the current state of the evidence base for use of RTG for nocturnal sleep problems in PD. Firstly, we have attempted to summarize the current understanding of some nocturnal sleep disturbances in PD and the rationale for the of RTG transdermal patch in this setting. A more detailed description of studies addressing specific features of sleep disruption and the use of RTG follows. We searched MEDLINE, EMBASE, and PsycINFO from inception through to October 15, 2016 using a combination of the following search terms: Parkinson* AND rotigotine AND sleep. The literature search was limited to open-label and blinded studies in the English language. To ensure a comprehensive coverage of the potential effects of RTG on sleep, studies addressing nocturnal pain and RBD were also identified through cited references and authors’ databases.

### Night-time sleep disturbances in Parkinson’s disease

Nocturnal sleep related problems in PD encompass a wide range of disturbances such as insomnia, RBD, nocturia, RLS, PLMS and sleep disordered breathing (SDB) that are often associated with one another.^1^ One of the most frequent reported however in PD is sleep fragmentation.^8, 9^ In community-based studies, frequent night-time awakenings were shown to occur in nearly 40% of patients.^10, 11^ Sleep fragmentation is clinically defined as the presence of recurrent, involuntary and frequent nocturnal awakenings (conscious arousals) that interrupt normal sleep maintenance and prevent patients from getting a normal amount of deep sleep. It is one of the earliest sleep problems to develop in PD patients, increasing in frequency with disease duration and motor stage.^8, 10^

The high frequency of sleep fragmentation is partly explained by its many potential etiologies, including nocturnal recurrence of motor symptoms, pain, restless legs-like symptoms, coexistent SDB, depression, or nocturia.^10, 12^ In one study, higher levodopa equivalent daily dose of levodopa preparations and dopamine agonists together with more severe depressive symptoms, better cognition and more severe motor fluctuations were shown to account for almost one third of the night-time sleep problems variance.^13^ An association with the type of dopamine agonist was not mentioned in the manuscript, yet, in clinical practice, potential drug induced sleep problems (including dopamine agonists) have always to be considered when approaching PD patients with insomnia. In addition, sleep disturbances have increasingly been considered as an inherent component of the degenerative process itself, associated with neuronal degeneration and both α-synuclein and tau deposition in key structures involved in sleep cycle and maintenance, such as the locus coeruleus, raphe nuclei, paramammilary and posterior hypothalamic nuclei, amygdala, and thalamus.^14^ More recently, the basal ganglia and dopaminergic pathways have implicated and attempts to describe its role and the effect of dopamine agonists have been made.^15–17^

Sleep fragmentation is not only an early, frequent, and disabling NMS in PD.^8–10^ The deleterious consequences of chronic sleep fragmentation have been seen to be numerous. Relationships between cognition and sleep are not only restricted to the presence of RBD.^18, 19^ Chronic sleep fragmentation has been associated with impairments in executive functions, including deficits in attention, phonemic verbal fluency, and working memory.^20, 21^ Moreover, the increase in the number of total nocturnal awakenings during PD progression correlates with a reduction in the percentage of slow-wave sleep,^22^ which is essential for executive performance,^20^ and memory consolidation.^23, 24^ Sleep fragmentation, by impacting on global sleep quality and sleep efficiency, has also been linked to stress-induced neurotoxic effects^25, 26^ and, recently, to defective functionality of the glymphatic system,^27^ with consequent higher cortical deposition of β-amyloid^28^ and insoluble tau protein.^29^

RLS in PD can further contribute to poor night-time sleep by potentially promoting hyperarousal episodes. Much uncertainty still exists about the relationship between RLS and PD, however several studies suggest a potential increased prevalence of RLS in PD patients compared to healthy controls.^30–34^ Problems arise from potential major confounders between PD and RLS and some authors postulate a secondary pathogenesis related to the use of dopaminergic therapy.^31^ In clinical practice, the diagnosis of RLS in PD follows the four essential diagnostic criteria proposed by the International RLS Study Group in 2003,^35^ with an emphasis on the “urge to move”, rest-induced and movement-responsive disorder; the diagnostic criteria have been revised not long ago in 2014.^36^ Sleep disruption is one of the primary factors producing morbidity in patients with moderate to severe idiopathic RLS,^37^ still the neurobiological basis of RLS is poorly understood. Studies have implicated the role of glutamatergic^38^ and dopaminergic neurotransmission,^39^ adenosine receptors,^40^ and their relation to brain iron deficiency, arguing for a multiple therapy approach.^41^ Recent published guidelines for the treatment of primary RLS have summarized the levels of evidence of several pharmacological and non-pharmacological treatments targeting different symptoms in RLS; when subjective sleep outcomes were used and when PLMS was the main target, studies have shown RTG as having moderate evidence in RLS.^42^

As mentioned previously, nocturia is also one of the main causes of sleep fragmentation among PD patients.^8^ The neurobiology of lower urinary tract symptoms in PD is complex, however, it has been shown that detrusor overactivity is the one of the major contributing factors for storage symptoms in PD, such as nocturia.^43^ Moreover, it has been thought that the net effect of the basal ganglia on micturition is inhibitory.^44^ Some studies have reported a storage-facilitating effect of some dopaminergic drugs, namely those with D1 activity, arguing that the frontal-basal D1 dopaminergic circuit that normally suppresses the micturition reflex is disrupted in PD.^44^ Although the effect of dopaminergic drugs on nocturia is far from being extensively investigated, these reports suggest potential differences among dopamine agonists when it comes to the treatment of nocturia in PD.

The reported prevalence of pain in PD patients varies depending on methodological assessments and definitions used; a recent systematic review indicated a mean pain prevalence of 68%.^45, 46^ Different types of pain have been recognized in PD, including musculoskeletal, dystonic, neuropathic, and central,^45, 47^ which further contribute to the complexity of the management of this NMS. A systematic review and meta-analysis of experimental studies has emphasized the potential role of dopaminergic neurotransmission and the role of dopaminergic drugs targeting central pain in PD patients,^48^ and the use of validated scales of pain in PD might help to further differentiate and address different pain syndromes in clinical practice.^47^

Despite the overwhelming evidence of night-time sleep disruption in PD and the different components that contribute to a bad quality of sleep, a recent systematic review and meta-analysis of all randomized trials comparing pharmacological interventions has highlighted the lack of evidence supporting specific interventions.^49^ In essence, the management of sleep dysfunction in PD remains complex and, in many cases, a key unmet need.

### Rotigotine pharmacology and why it may help with sleep related problems in Parkinson’s disease

RTG (previously N-0923) is a non-ergolinic aminotetralin dopamine agonist delivered using a silicone-based transdermal patch.^19^ The transdermal formulation has proven to be useful due to RTG’s extensive first-pass gastrointestinal metabolism, short elimination half-life after intravenous administration, and high lipid soluble properties.^50–52^ The important advantages of a 24-hour delivery system, bypassing the gastrointestinal (GI) system and a once daily application have been previously described.^53^ Caregivers’ perceived advantages over oral therapies have also been highlighted recently, adding a much needed perspective in PD management.^54^

RTG is a D3/D2/D1 dopamine agonist with antagonist activity at α2B receptors and weak, but significant, agonistic activity at 5-HT1A.^19, 55, 56^ Analysis of three studies in healthy subjects (SP 871) and early PD (SP630, SP 651) confirm the sustained and steady maintenance of RTG plasma levels overnight with a continuous transdermal delivery system.^57^ Moreover, together with apomorphine, RTG is one of the few dopamine agonists extensively used in clinical practice that has significant effect on D1-type receptors.

PD across all motor stages is associated with upper GI dysfunction such as delayed gastric emptying^58^ and comorbidities such as *Helicobacter pylori* infection.^59^ The case for non-oral routes delivery mechanisms, like the transdermal patch, are being increasingly recognized as a cogent choice for management of motor and NMS.^60^ Recent evidence suggests that EMO periods are highly prevalent in treated PD^6^ and are likely to be related to delayed gastric emptying and consequent poor absorption of oral levodopa.^60^ This observation is supported by the fact that RTG and non-oral therapies can overcome EMO symptoms.^7, 61, 62^ Furthermore, more long-acting dopamine receptors like ropinirole prolonged release and cabergoline have all been shown to improve sleep by significant reductions of motor PD symptoms at night.^63, 64^

It is reasonable to assume that the non-oral route and the continuous stimulation achieved by RTG patch would be helpful for several aspects of sleep dysfunction in PD as outlined in Table 1 and further described in the following sections. Moreover, the RTG’s specific effects on different types dopamine receptors and others, might further contribute to its potential beneficial effects on nocturnal sleep disruption in PD, although this remains speculative. In the following sections we discuss open-label and blinded studies investigating the effects of RTG in night-time sleep disturbances.

**Table 1:** List of sleep related dysfunction/abnormality in PD where use of RTG transdermal patch may be helpful

Sleep Symptoms in PD	Nature of trial	RTG dose^a^	Sleep-related important findings
Nocturnal motor symptoms, sleep fragmentation, pain	Single center, open-label, single-arm study^28^	2–4 mg/day (overnight)	Improvement of quality of nocturnal sleep and difficulty in staying asleep (PDSS individual items, both *p* < 0.05) in advanced PD (14+ years)
Nocturnal sleep disturbances, nocturia	Single center, open-label, single-arm study^29^	11.8 ± 3.9 mg/24 h	Overall improvement in sleep quality (PDSS total score)
			Decrease of number of nocturias [from 2.05 (0–6) to 1.4 (0–3.5) counts per night]
Sleep quality, nocturia	German multicenter, open-label, single-arm study^30^	6.6 ± 2.5 mg/24 h	Improvement of sleep quality and nocturia (VAS)
Early morning motor symptoms, nocturnal sleep disturbance	Multinational, multicenter, double-blind, randomized, placebo-controlled (RECOVER) study^9^	2–16 mg/24 h	All 15 individual PDSS-2 items except ‘distressing hallucinations’ showed significant improvements, particularly ‘difficulty falling asleep’, ‘urge to move arms or legs’ and ‘uncomfortable or immobile’ (*p* < 0.001)
Early morning motor symptoms, nocturnal sleep disturbance	Open-label extension of the RECOVER study^31^	11.5 ± 3.8 mg/24 h	Stable improvement in sleep seen over a period of up to 1 year (PDSS-2)
Sleep fragmentation, nocturnal motor symptoms, RLLS, nocturia	Spanish, multicenter, open-label, single-arm (SLEEP-FRAM) study^32^	8.5 ± 3.0 mg/24 h	Improvement of sleep fragmentation (PDSS-2, *p* < 0.0001), nocturnal motor symptoms (*p* < 0.0001), RLLS (*p* < 0.005) and nocturia (*p* < 0.004)
Nocturnal sleep disturbances, nocturia, pain	Spanish, multicenter, open-label, single arm study^33^	11.8 mg/day (overnight)	Nocturia as a major complain in 69.1% of patients at baseline
			Overall improvement in sleep quality (PDSS-2 total score, *p* < 0.001)
			Improvement of pain (VAS Pain, *p* < 0.001)
Sleep fragmentation, RLLS, nocturia	Single-center, open-label, single-arm study^34^	6–8 mg/24 h	Amelioration of sleep maintenance (PDSS-2, *p* = 0.018), uncomfortable sensation due to immobility (*p* = 0.011), RLLS (*p* = 0.026), and nocturia (*p* = 0.04)
			Improvements in WASO (actigraphy, *p* = 0.013), SE (*p* = 0.017), mean duration of wake episodes (*p* = 0.005)
Nocturnal sleep disturbances, sleep fragmentation, PLMS	Single-center, open-label, single-arm study^35^	10.56 ± 6.34 mg/24 h	Overall improvement in sleep quality (PDSS-2 total score, *p* < 0.011)
			Improvement of SE (VPSG, *p* = 0.034), SL (*p* = 0.044), WASO (*p* = 0.048), and PLMS index (*p* = 0.000)
Nocturnal sleep disturbances, sleep fragmentation	Single-center, double-blind, randomized, placebo-controlled study^36^	9.14 ± 1.85 mg/day (overnight)	Overall improvement in sleep quality (PDSS-2 total score and PSQI, *p* < 0.01)
			Improvement of SE (PSG, *p* < 0.001), WASO (*p* < 0.001), SL (*p* < 0.001)
Nocturnal pain	Multinational, multicenter, double-blind, randomized, placebo-controlled study^37^	14.7 ± 5.1 mg/24 h	No statistically significant improvement in KPPD, including nocturnal pain
			Improvement in quality of life (PDQ-8, *p* = 0.038)

*KPPD*King’s PD pain scale, *PD* Parkinson’s disease, *PDSS* Parkinson’s disease sleep scale, *PDSS-2* Parkinson’s disease sleep scale 2, *PMLS* periodic limb movement of sleep, *RBD* rapid eye movement sleep behavior disorder, *RLLS* restless legs-like symptoms, *RTG* rotigotine, *SE* sleep efficiency, *SL* sleep latency, *VAS* visual analog scale, *WASO* wake after sleep onset

^a^ As most presented studies were designed in a pragmatic fashion, the wide range of RTG dosages used might reflect different PD population groups regarding disease duration, concomitant dopaminergic therapy, side-effects or local standard clinical practice

### Sleep fragmentation

**Fig. 1:**
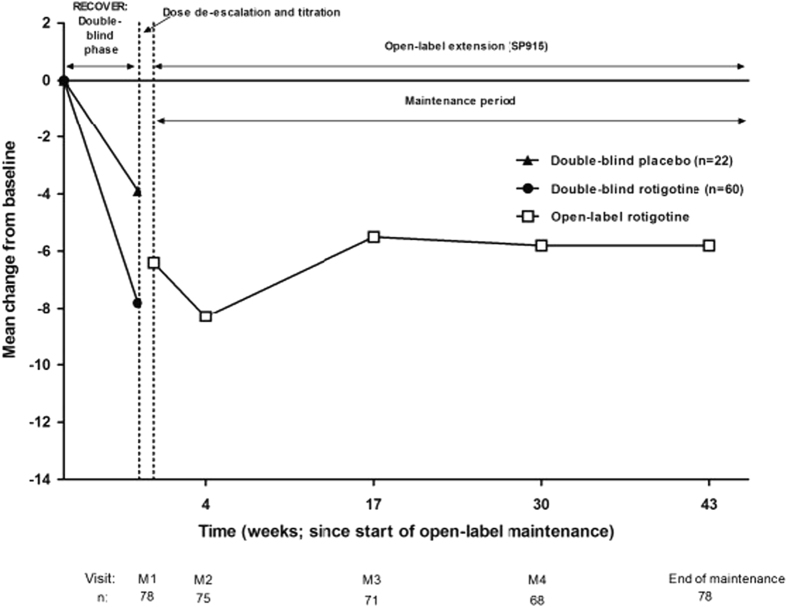
Mean change from double-blind baseline in PDSS-2 scores over time from baseline of RECOVER to the end of maintenance of the open-label extension (mean change scores of those subjects who subsequently enrolled in the open-label extension are shown by double-blind randomization [placebo or RTG] during the double-blind [RECOVER] phase and for the combined study population in the open-label phase).^65^ Reproduced with permission (https://creativecommons.org/licenses/by-nc-nd/3.0/) from ref. 65 ^©^(2012) Elsevier

No specific treatment for sleep fragmentation is currently established in clinical guidelines for PD, but some of its causes can be treated by the use of long-acting dopaminergic agents. The multinational, double-blinded, randomized, placebo-controlled RECOVER study investigated the effects of RTG in a sample of 246 PD patients with unsatisfactory early-morning motor symptom control.^7^ Nocturnal sleep disturbance measured by PDSS-2 was assessed as a coprimary endpoint. RTG transdermal patch (*n* = 191; 2–16 mg/24 h; mean age 64.8 ± 9.3 years, 64% male) compared to placebo (*n* = 96; mean age 64.4 ± 10.6 years; 64% male) significantly improved motor function, early morning akinesia, and nocturnal sleep disturbances in patients with PD (PDSS-2 treatment difference −4.26 [95% CI −6.08, −2.45]; *p* < 0.0001). Moreover, the reported beneficial effects of RTG patch have been shown to be sustained in a 1 year open label extension of that study as shown in Fig. 1.^65^ However, regarding individual items of the PDSS-2, specifically individual item 3 (‘Did you have difficulty staying asleep?’), no significant improvement was observed in the original RECOVER study.^7^ Whereas patients receiving RTG or placebo showed no significant differences in PDSS-2 total score at baseline, it is not clear whether patients with sleep maintenance problems were well-balanced between treatment arms. Considering the limitations of the study and the importance of specifically assessing whether RTG could be effective for improving sleep fragmentation, posterior studies were performed including patients with self-reported complaints of recurrent nocturnal awakenings.

The multicenter prospective SLEEP-FRAM study explored the effects of RTG on sleep disruption in a sample of 62 PD patients (mean age 70.2 ± 7 years; 66% male; mean Hoehn and Yahr stage (HY) 2.2 ± 0.8) with self-reported complaints of nocturnal awakenings, but well-controlled parkinsonian motor symptoms during daytime.^66^ The main primary endpoint was a change from baseline in sleep fragmentation, as assessed by the item 3 of the PDSS-2. Secondary outcome variables included a small number of PDSS-2 subscale scores and other self-reported sleep measures. After 3 months of treatment, RTG (mean dose 8.9 mg/day) significantly improved sleep fragmentation [from 3.4 ± 0.9 to 1.9 ± 1.4 (*p* < 0.0001)]; patients improved from getting up at night 4–5 times to twice per week. By analyzing the different nocturnal symptoms explored by the PDSS-2, improvement in sleep fragmentation was driven by significant improvement of parkinsonian nocturnal motor symptoms (e.g., difficulties turning around in bed, rigidity, muscle cramps), restless-legs like symptoms and nocturia. This study had some important limitations. It was an open-label trial with no control group and the primary outcome was solely based on subjective reports. Moreover, patients were followed up for only three months and other sleep problems that can only be assessed using polysomnography (SDB, RBD, PLMS), were not analyzed. Correction for multiple testing was not mentioned in the manuscript, however, the study was restricted to a small number of planned comparisons clearly described in the protocol.

A more objective improvement of RTG on sleep fragmentation was observed in another open-label study that included 15 PD patients (mean age 67 ± 9 years; 80% males; mean disease duration 5 ± 3 years) with self-reported sleep complaints and a PDSS-2 total score ≥ 10.^67^ In stable PD patients with HY < 3, the effect of RTG (6 – 8 mg/day) on different sleep parameters was assessed by sleep diaries, the PDSS-2, and actigraphic recordings at baseline and after 2 months of treatment. Similarly to previous studies, RTG significantly improved PDSS-2 total score [from 20 (16–30) to 9 (4–20); *p* = 0.001], which correlated with significant improvement in the PD Questionnaire-39 (PDQ-39) total score (*p* = 0.016). More specifically, the PDSS-2 symptoms that improved were very similar to those found in the SLEEP-FRAM study, with amelioration of sleep maintenance, uncomfortable sensation due to immobility, restless-legs like symptoms (limb restlessness, urge to move limbs), and nocturia. According to patients’ diaries, nights of good sleep per week significantly increased, and number and duration of nocturnal awakenings significantly decreased. In the actigraphic recordings, improvements were observed in Wake-time After Sleep Onset (WASO; *p* = 0.013), sleep efficiency (total sleep time /time in bed; *p* = 0.017), and mean duration of wake episodes (*p* = 0.005), which patients often report as most bothersome. Besides the open-label nature of the study and the lack of a control group, other important limitations include the small sample size and the short period of follow-up. Additionally, specific primary outcomes on sleep were not clearly defined in the protocol, and a number of self-reported sleep and nocturnal actigraphic measures were used to assess potential beneficial effects of RTG on sleep disturbance. Statistical significance was set at *p* ≤0 .05 and a rationale for the potential adjustment of *p* values was not stated.

Objective improvements of RTG were also evaluated in another open-label study in a sample of 25 PD patients (mean age 63.12 ± 12.21 years; 64% males; mean HY 2.58 ± 0.94) with self-reported unsatisfactory nocturnal sleep disturbances.^68^ In addition to subjective sleep measures (PDSS-2 total score for nocturnal sleep; Epworth Sleepiness Scale (ESS) for daytime sleepiness), participants underwent overnight video-polysomnography (VPSG) tests. This allowed objective measurements of sleep quality at baseline and at the end of the up to 6 months dose-maintenance period (RTG daily dose 10.56 ± 6.34 mg; mean time of RTG therapy 20.6 ± 8.08 weeks). The significant improvement in the PDSS-2 observed was small [from baseline 19.96 ± 8.51 to end of treatment 18.32 ± 7.83; *p* = 0.011] but in line with previous studies showing an overall improvement, and the worsening of ESS total score was found not to be significant [from baseline 4.72 ± 2.99, end of treatment 6.24 ± 4.00; *p* = 0.077]. More importantly, sleep efficiency was significantly increased [from 68.59 ± 16.06 to 74.04 ± 15.39%, *p* = 0.034], while the sleep latency [from 28.28 ± 32.56 to 18.08 ± 13.14 min; *p* = 0.044], WASO [from 14.44 ± 8.68 to 10.76 ± 6.08 times; *p* = 0.048], and PLMS index [from 22.68 ± 18.97 to 14.24 ± 14.58; *p* = 0.000] were significantly decreased after RTG treatment. Improvements of specific individual items of the PDSS-2 and the mean duration of wake episodes were not mentioned in the manuscript. Limitations of this study include some of the above mentioned shortcomings such as the open-label design, lack of a control group and the small sample size. However, despite its exploratory nature, the main outcome variables were defined in the protocol and restricted to a limited number of subjective and objective endpoint parameters; statistical significance was set up at *p* < 0.05.

Open-label studies limitations have been overcome by a recent double-blinded, randomized, placebo-controlled study that have assessed the efficacy of RTG on sleep fragmentation in PD patients, by using subjective sleep scales (PDSS-2, Pittsburgh Sleep Quality Index) and polysomnography.^69^ The study enrolled 42 patients that received either RTG (*n* = 21; 9.14 ± 1.85 mg/day; mean age 63.28 ± 2.98 years; mean HY 2.28 ± 0.25) or placebo (*n* = 21; mean age 64.04 ± 2.90 years; mean HY 2.23 ± 0.25) during 10 weeks. RTG patches were administered from 18:00 h to awakening, in order to minimize the influence that RTG improvement on diurnal motor symptoms may have on sleep parameters. At the end of the study, both PDSS-2 and PSQI total scores improved significantly (*p* < 0.01), but the authors did not describe which particular PDSS-2 individual items improved the most. Regarding the primary outcome, changes in polysomnography, RTG significantly improved sleep efficiency (*p* < 0.001), WASO (*p* < 0.001), as well as sleep latency (*p* < 0.001) compared to baseline and the placebo group; *p*-values were compensated by the Bonferroni correction for multiple comparisons. Mean duration of wake episodes was not measured. It is remarkable–both for clinical and research purposes–that clear and significant correlations were shown between scores in subjective sleep assessment tools and polysomnographic parameters, suggesting that the use of the PDSS-2 seems appropriate for assessing changes in sleep when polysomnography is not available.

### Restless legs syndrome

**Fig. 2:**
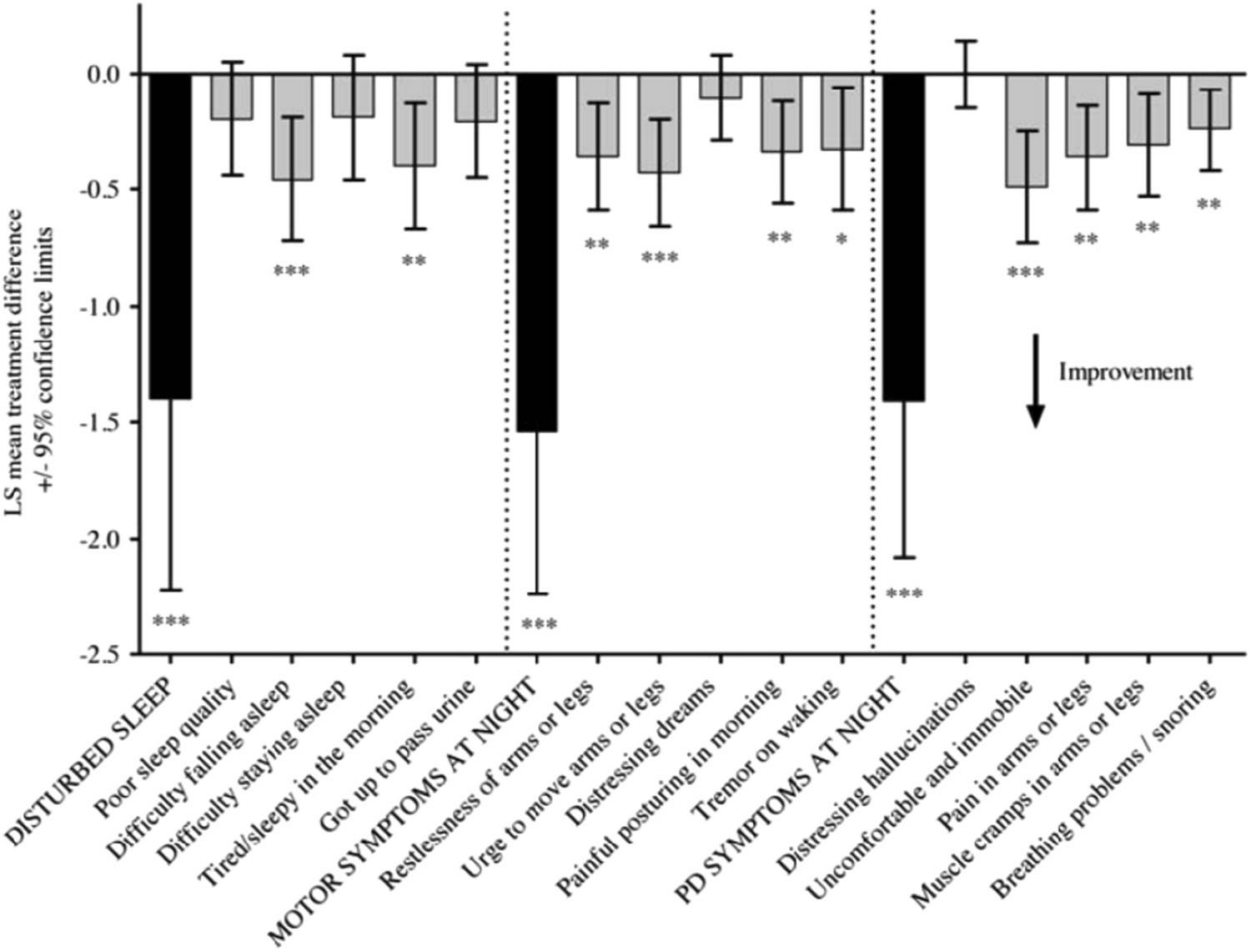
PDSS-2 domain and individual items related to RLS show differences between RTG treatment and placebo arms (LS = least squares; *p < 0.05; **p < 0.01; ***p < 0.001 for rotigotine-placebo treatment difference).^7^ Reproduced with permission from ref. 7 ^©^(2011) Wiley

There are currently no trials assessing the efficacy of RTG with RLS in PD as a primary outcome measure. Extrapolation of data from the RECOVER study allowed analysis of the PDSS-2 (which has RLS related questions under ‘Motor symptoms at night’ domain) which suggested that the two items of the PDSS-2 related to RLS (‘Restlessness of arms and legs’ and ‘Urge to move arms or legs’, as shown in Fig. 2) are significantly improved by application of RTG patch compared to placebo (*p* < 0.01 and *p* < 0.001, respectively).^7^ Further studies have corroborated the potential beneficial effect of RTG on restless legs-like symptoms as assessed by the two PDSS-2 individual items related to RLS.^66, 67^ Furthermore, PLMS has also been shown to improved when assessed by the PMLS index in recently published open-label studies using VPSG recordings.^68, 70^

### Nocturia

In the SLEEP-FRAM study and in the study by Calandra–Buonaura and colleagues, patients with sleep complaints receiving RTG had significantly less nocturia when compared to baseline at 3-month and 2-month of follow-up, respectively (*p* = 0.04 in both studies), as assessed by the PDSS-2 individual item 8.^66, 67^

Another open-label single-arm study addressing the effect of night-time use of RTG in a sample of 54 PD patients (mean age 65.0 ± 10.0 years; 64.8% male; mean disease duration 5.25 ± 3.4 years) with self-reported unsatisfactory control of early morning motor impairment observed encouraging beneficial effects on nocturia as well.^71^ An improvement of the number of nocturias [from baseline to end of treatment of −0.66 ± 0.9] was noted after an up to 4 week dose-maintenance period (11.8 mg/24 h). An open-label study that selected 74 PD patients (mean age 71.5 ± 8.6 years; 54.3% male; mean disease duration 5.6 ± 5.2 years) with unsatisfactory control of nocturnal and early morning symptoms observed that nocturia was the major complaint of night-time sleep disturbance in two third of the patients.^72^ The authors also noted a significant improvement in all 3 domains of the PDSS-2, including nocturia, after 3 months of night-time RTG treatment (mean 5.31 mg/day) compared to baseline assessments.

An open-label study investigating the potential effects of RTG in combination therapy in a sample of 969 PD patients (age 70.0 ± 9.3 years; 61.7% male) used individual items 1 (‘The overall quality of your night’s sleep is’) and 8 (‘Do you get up at night to pass urine?’) from the PD Sleep Scale (PDSS) to assess sleep quality and nocturia.^73^ For each item, participants were given a visual analog 100 mm scale from 0 (worst state) to 100 (best state) and were asked to rate their respective state at baseline and at two follow up visits, 5 to 10 weeks and 12 to 16 weeks following RTG initiation, respectively (5.8 ± 2.3 mg/24 h and 6.6 ± 2.5 mg/). Eligible participants did not need to have specific sleep problems to be enrolled as the primary objective of the study was to evaluate RTG administration in combination with other antiparkinsonian medication in routine clinical practice. Both items assessing sleep quality and nocturia continuously improved during RTG treatment, and the occurrence of nocturias showed an increase by 13.4 points from baseline to the last follow up visit in the above mentioned analog scale.

Despite the evidence from open-label studies, these positive results were not replicated in the RECOVER study,^7^ where no significant improvement of nocturia could be found in the RTG treated group when compared to placebo as assessed by the PDSS-2 individual item 8 (‘Got up to pass urine’). The NMS Scale (NMSS) score used as a secondary outcome measure to address nocturia, did not show a significant improvement when comparing the RTG treated and the placebo-controlled group. The reasons for the discrepancy remain unknown, however the RECOVER study was designed to specifically address early morning symptoms, not including potential patients without EMO but with significant night-time sleep disturbance associated with nocturia.

### Nocturnal pain

Pain poses a big burden on affected PD patients and has been reported by as many as one third of the patients included in the RECOVER study.^7^ The effect of RTG treatment on pain has been assessed by a post-hoc analysis of the RECOVER study^74^ by means of the Likert pain scale, as well as analyzing pain in other scales including Nocturnal Akinesia Dystonia and Cramp Score (NADCS), PDQ-8, PDSS-2 and NMSS. The results showed a significant improvement in pain in the subgroup of moderate to severe pain (pain score above 3 points in the Likert pain scale) when compared to placebo, but not in the subgroup of mild pain (1–3 points in the Likert pain scale).

A double-blinded, randomized, placebo-controlled study in a sample of 60 patients further investigated the effect of the RTG patch on PD-associated pain as a primary outcome.^75^ RTG transdermal patch (*n* = 35; 14.7 ± 5.1 mg/24 h; age 66.5 ± 11.9 years; 54% male [safety set]) compared to placebo (*n* = 33; age 65.3 ± 13.8 years; 52% male [safety set]) did not show statistically significant improvements in the King’s PD Pain Scale (KPPS). However, when assessing the changes in pain in the PDQ-8 score in the RTG and the placebo arms, significance levels were reached, postulating a possible improvement of pain by RTG therapy.

Moreover, improvement of pain by RTG treatment has also been reported by an open-label study.^72^ Pain, as assessed by the visual analog scale (VAS) score, significantly improved after 3 months of exclusively night-time RTG treatment (mean 5.31 mg/day) when compared to baseline [from 3.2 ± 2.5 to 2.3 ± 2.4; *p* < 0.001] in a group of 74 patients.

### REM sleep behavior disorder

While the pathophysiological origin of RBD is unclear and likely to be non-dopaminergic, one study from China performed an open label study in 11 PD patients using a RBD Questionnaire-Hong Kong (RBDQ-HK) and blinded VPSG assessments.^70^ The authors reported a subjective improvement of the motor aspects of RBD after RTG along with improvement in PLMS index and total sleep time, although RBD related sleep parameters were unaffected. The role of RTG in the management of RBD therefore, needs to be explored.

### Rotigotine in apomorphine or other advanced therapies treated advanced Parkinson’s patients

The effects of RTG on sleep disturbances in PD are most frequently assessed in groups of patients with a fairly early disease course, however the described potential beneficial effects of RTG might be observed in advanced PD patients as well. An open-label single-arm study in a small sample of 6 PD patients (age 60.4 ± 7.8 years; 83.3% male; disease duration 17.4 ± 3.2 years) investigated the effects of RTG patch on sleep disorders in advanced PD treated with day-time apomorphine infusion (3.1 ± 0.8 mg/h for 12 h).^76^ Compared to baseline, the PDSS total score decreased on average 44.8% [from 21.2 ± 2.8 to 11.7 ± 2.8; *p* < 0.05] at 4-month follow up. Improvements in 12 out of the 15 items of the scale were recorded, with quality of sleep and difficulty in staying asleep showing statistically significant differences (both *p* < 0.05). Furthermore, the VAS score used to quantify sleep problems improved by 65% [from 2.6 ± 0.6 to 7.4 ± 0.6]. This small-open label study supports the use of low-dosage RTG in advanced PD patients with sleep disturbances, indicating a good tolerance with no incidence of relevant side effect such as hallucinations. Further studies addressing the effect and tolerability of RTG on sleep disturbances in advanced PD patients are needed to confirm these preliminary observations.

### Possible adverse sleep related events

Most studies do not mention any specific sleep related side effects of RTG use and it seems well tolerated.^66, 69, 71–73, 75, 76^ In the RECOVER study, two RTG treated subjects reported a sleep attack and one participant had suggestive findings of compulsive sexual behavior.^7^

An open-label study investigated the Clinical Global Impression item 4 (CGI-4) assessing safety as a primary outcome of RTG as an add-on to oral dopamine agonist therapy.^77^ In a sample of 79 PD patients (age 61.3 ± 9.3 years; 52% male; disease duration 7.4 ± 3.9 years) with EMO or nocturnal sleep disturbance, the RTG maintenance dose was 5.71 ± 2.28 mg/24 h for 58.7 ± 14.9 days, while the concomitant oral dopamine agonist (pramipexole 57%, ropinirole 43%) as RTG converted dose at baseline was 4.02 ± 1.66 mg/24 h. The authors noted an adverse event profile similar to previous studies of RTG in patients with advanced PD. Hallucinations were observed in two subjects, and somnolence, insomnia and impulse compulsive behavior in three subjects. Furthermore, no obvious relationship with total dopamine agonist dose was found. Improvements in the PDSS-2 total score, PSQI global score, and PDSS-2 individual items relating to sleep maintenance, while worsening of item 7 (distressing hallucinations at night) were also observed. Most importantly, 93% of the subjects showed a CGI-4 score < 3 indicating that the add-on therapy did not interfere with functioning. Besides the above mentioned nocturnal hallucinations in two subjects and insomnia in one patient, no other sleep related events were reported.

Other open-label studies have observed a decrease in number or duration of daytime sleep episodes with RTG therapy ^67^ or no significant increase in day-time somnolence.^68^

While other dopamine agonists may have variable effects on sleep architecture and thus insomnia, RTG therapy, as noted in the selected studies, did not appear to have any adverse events related to insomnia or indeed there are no reports of RTG related nocturnal binge eating as a manifestation of impulse control disorder. Furthermore, preliminary studies also suggest that add-on RTG to other dopamine agonist therapy is well tolerated in most patients.

## Conclusion

Original evidence for the use of RTG for nocturnal dysfunction in PD has been suggested in several open-label studies, which have been eluded to in this review and, importantly, in the RECOVER study which was the first one to use the PDSS-2 as a core primary outcome measure. The data were extremely powerful, suggesting a strong effect of RTG transdermal patch on several aspects of sleep problems in PD, particularly around maintenance of sleep, nocturnal restlessness, nocturnal akinesia, as well as sleep refreshment. At the same time, it showed that the RTG transdermal patch did not aggravate daytime somnolence as the rates were similar to that of placebo. These observations were confirmed in a post hoc analysis that was published based on the RECOVER study data. Furthermore, subsequently published studies have overall confirmed the potential beneficial effects of RTG transdermal patch on sleep dysfunction in PD, including nocturia, one of the most frequent and distressing symptoms reported as shown by the NIGHT-PD study.^8, 78^

While clinically, sleep dysfunction in PD can be evaluated by the PDSS, now widely used and validated across the globe, the effect of dopaminergic drugs on sleep architecture is rather more complex. Subjective sleep complaints are particularly sensitive to a placebo effect and most studies discussed have not used objective sleep parameters such as polysomnography. However, available evidence suggests that the RTG transdermal has a sustained benefit on sleep fragmentation and sleep efficiency as well.

### Suggested specific clinical uses

**Fig. 3:**
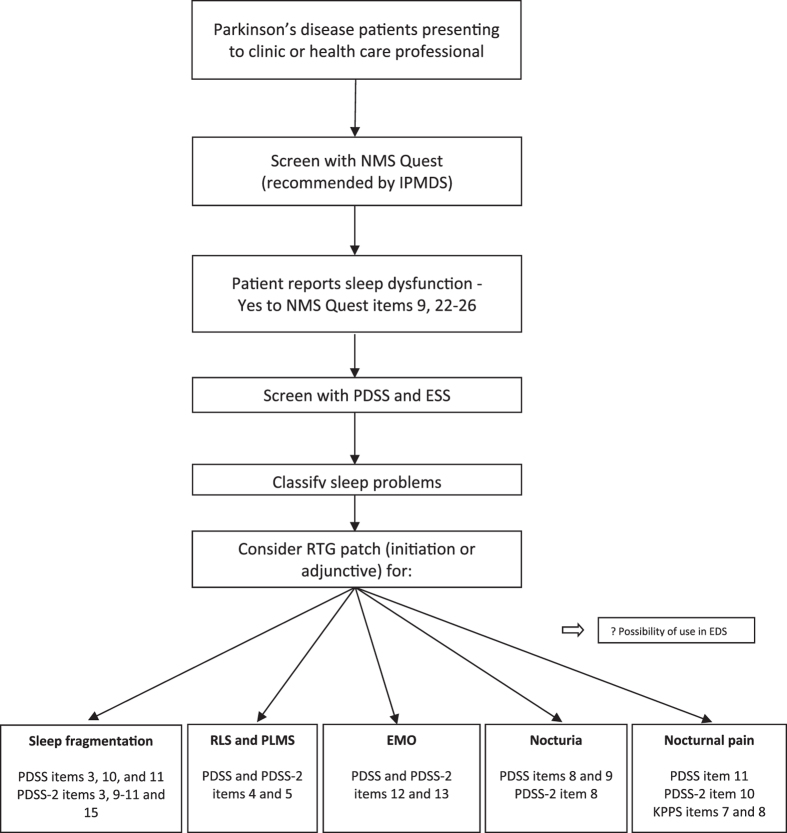
Individualized medicine and rotigotine. *EMO* early morning off, *ESS* Epworth sleepiness scale, *IPMDS* International Parkinson and Movement Disorder Society, *KPPS* King’s Parkinson’s Pain Scale, *NMSQuest* non-motor symptoms questionnaire, *PDSS* Parkinson’s disease sleep scale, *PDSS-2* Parkinson’s disease sleep scale 2, *PLMS* periodic limb movements in sleep, *RLS* restless legs syndrome, *RTG* rotigotine

Taken all this into consideration, one would therefore be inclined to suggest that currently the evidence base substantiate that, if a PD patient reports sleep problems, which can be further defined and delineated by using the PDSS, RTG transdermal patch may be a good treatment alternative (Fig. 3). Obviously, one has to be aware that the neurobiology of sleep dysfunction in PD is complex and not only dopaminergic in nature, and therefore, we do not envisage the use of RTG transdermal patch for all sleep problems in PD.

Further large scale studies, in a controlled fashion using objective measures of sleep in PD, preferably with a multicenter design, would therefore be welcome to confirm the efficacy of RTG transdermal patch and sleep dysfunction in PD.

## References

[1] Chahine, L. M., Amara, A. W. & Videnovic, A. A systematic review of the literature on disorders of sleep and wakefulness in Parkinson's disease from 2005 to 2015. *Sleep Med. Rev.* doi:10.1016/j.smrv.2016.08.001 (2016). 10.1016/j.smrv.2016.08.001 PMC5332351 27863901

[2] Chaudhuri KR, Healy DG, Schapira AH (2006). Non-motor symptoms of Parkinson's disease: diagnosis and management. Lancet Neurol..

[3] Berg D (2015). MDS research criteria for prodromal Parkinson's disease. Mov. Disord..

[4] Postuma RB (2016). Screening for prodromal Parkinson's disease in the general community: a sleep-based approach. Sleep Med..

[5] Happe S, Trenkwalder C (2003). Movement disorders in sleep: Parkinson's disease and restless legs syndrome. Biomed. Technik. Biomed. Eng..

[6] Rizos A (2014). Characterizing motor and non-motor aspects of early-morning off periods in Parkinson's disease: an international multicenter study. Parkinsonism Relat. Disord..

[7] Trenkwalder C (2011). Rotigotine effects on early morning motor function and sleep in Parkinson's disease: a double-blind, randomized, placebo-controlled study (RECOVER). Mov. Disord..

[8] Porter B, Macfarlane R, Walker R (2008). The frequency and nature of sleep disorders in a community-based population of patients with Parkinson's disease. Eur. J. Neurol..

[9] Norlinah MI (2009). Sleep disturbances in Malaysian patients with Parkinson's disease using polysomnography and PDSS. Parkinsonism Relat. Disord..

[10] Gjerstad MD, Wentzel-Larsen T, Aarsland D, Larsen JP (2007). Insomnia in Parkinson's disease: frequency and progression over time. J. Neurol. Neurosurgery Psychiatry.

[11] Tandberg E, Larsen JP, Karlsen K (1998). A community-based study of sleep disorders in patients with Parkinson's disease. Mov. Disord..

[12] Comella CL (2007). Sleep disorders in Parkinson's disease: an overview. Mov. Disord..

[13] Verbaan D, van Rooden SM, Visser M, Marinus J, van Hilten JJ (2008). Night time sleep problems and daytime sleepiness in Parkinson's disease. Mov. Disord..

[14] Kalaitzakis ME, Gentleman SM, Pearce RK (2013). Disturbed sleep in Parkinson's disease: anatomical and pathological correlates. Neuropathol. Appl. Neurobiol..

[15] French IT, Muthusamy KA (2016). A review of sleep and its disorders in patients with parkinson's disease in relation to various brain structures. Front. Aging Neurosci..

[16] Lazarus M, Chen JF, Urade Y, Huang ZL (2013). Role of the basal ganglia in the control of sleep and wakefulness. Curr. Opinion Neurobiol..

[17] Dauvilliers Y, Tafti M, Landolt HP (2015). Catechol-O-methyltransferase, dopamine, and sleep-wake regulation. Sleep Med. Rev..

[18] Erro R (2012). Link between non-motor symptoms and cognitive dysfunctions in de novo, drug-naive PD patients. J. Neurol..

[19] Jenner P (2005). A novel dopamine agonist for the transdermal treatment of Parkinson's disease. Neurology.

[20] Scullin MK, Trotti LM, Wilson AG, Greer SA, Bliwise DL (2012). Nocturnal sleep enhances working memory training in Parkinson's disease but not Lewy body dementia. Brain.

[21] Stavitsky K, Neargarder S, Bogdanova Y, McNamara P, Cronin-Golomb A (2012). The impact of sleep quality on cognitive functioning in Parkinson's disease. J. Int. Neuropsychol. Soc..

[22] Diederich NJ, Paolini V, Vaillant M (2009). Slow wave sleep and dopaminergic treatment in Parkinson's disease: a polysomnographic study. Acta Neurol. Scand..

[23] Stickgold R (2005). Sleep-dependent memory consolidation. Nature.

[24] Wilson MA, McNaughton BL (1994). Reactivation of hippocampal ensemble memories during sleep. Science.

[25] Banks S, Dinges DF (2007). Behavioral and physiological consequences of sleep restriction. J. Clin. Sleep Med..

[26] Sportiche N (2010). Sustained sleep fragmentation results in delayed changes in hippocampal-dependent cognitive function associated with reduced dentate gyrus neurogenesis. Neuroscience.

[27] Nedergaard M (2013). Neuroscience. Garbage truck of the brain. Science.

[28] Spira AP, Gamaldo AA, An Y (2013). Self-reported sleep and β-amyloid deposition in community-dwelling older adults. JAMA Neurol..

[29] Di Meco A, Joshi YB, Pratico D (2014). Sleep deprivation impairs memory, tau metabolism, and synaptic integrity of a mouse model of Alzheimer's disease with plaques and tangles. Neurobiol. Aging.

[30] Bhalsing K, Suresh K, Muthane UB, Pal PK (2013). Prevalence and profile of Restless Legs Syndrome in Parkinson's disease and other neurodegenerative disorders: a case-control study. Parkinsonism Relat. Disord..

[31] Angelini M, Negrotti A, Marchesi E, Bonavina G, Calzetti S (2011). A study of the prevalence of restless legs syndrome in previously untreated Parkinson's disease patients: absence of co-morbid association. J. Neurol. Sci..

[32] Krishnan PR, Bhatia M, Behari M (2003). Restless legs syndrome in Parkinson's disease: a case-controlled study. Mov. Disord..

[33] Rijsman RM, Schoolderman LF, Rundervoort RS, Louter M (2014). Restless legs syndrome in Parkinson's disease. Parkinsonism Relat. Disord..

[34] Dhawan V, Healy DG, Pal S, Chaudhuri KR (2006). Sleep-related problems of Parkinson's disease. Age Ageing.

[35] Allen RP (2003). Restless legs syndrome: diagnostic criteria, special considerations, and epidemiology. A report from the restless legs syndrome diagnosis and epidemiology workshop at the National Institutes of Health. Sleep Med..

[36] Allen RP (2014). Restless legs syndrome/Willis-Ekbom disease diagnostic criteria: updated International Restless Legs Syndrome Study Group (IRLSSG) consensus criteria--history, rationale, description, and significance. Sleep Med..

[37] Kushida CA, Allen RP, Atkinson MJ (2004). Modeling the causal relationships between symptoms associated with restless legs syndrome and the patient-reported impact of RLS. Sleep Med..

[38] Allen RP, Barker PB, Horska A, Earley CJ (2013). Thalamic glutamate/glutamine in restless legs syndrome: increased and related to disturbed sleep. Neurology.

[39] Earley CJ (2014). Altered Brain iron homeostasis and dopaminergic function in Restless Legs Syndrome (Willis–Ekbom Disease). Sleep Med..

[40] Quiroz C (2016). Adenosine receptors as markers of brain iron deficiency: Implications for Restless Legs Syndrome. Neuropharmacology.

[41] Ferré S, Earley C, Gulyani S, Garcia-Borreguero D (2017). In search of alternatives to dopaminergic ligands for the treatment of restless legs syndrome: iron, glutamate, and adenosine. Sleep Med..

[42] Winkelman JW (2016). Practice guideline summary: treatment of restless legs syndrome in adults: report of the guideline development, dissemination, and implementation subcommittee of the american academy of neurology. Neurology.

[43] Ruffion A (2013). Systematic review of the epidemiology of urinary incontinence and detrusor overactivity among patients with neurogenic overactive bladder. Neuroepidemiology.

[44] Sakakibara R (2014). Bladder function of patients with Parkinson's disease. Int. J. Urol..

[45] Young Blood MR, Ferro MM, Munhoz RP, Teive HA, Camargo CH (2016). Classification and characteristics of pain associated with parkinson's disease. Parkinson's Dis..

[46] Broen MP, Braaksma MM, Patijn J, Weber WE (2012). Prevalence of pain in Parkinson's disease: a systematic review using the modified QUADAS tool. Mov. Dis..

[47] Chaudhuri KR (2015). King's Parkinson's disease pain scale, the first scale for pain in PD: An international validation. Mov. Dis..

[48] Thompson T (2017). Pain perception in Parkinson’s disease: A systematic review and meta-analysis of experimental studies. Ageing Res. Rev..

[49] Rodrigues TM, Castro Caldas A, Ferreira JJ (2016). Pharmacological interventions for daytime sleepiness and sleep disorders in Parkinson's disease: Systematic review and meta-analysis. Parkinsonism Relat. Disord..

[50] Calabrese VP (1998). N-0923, a novel soluble dopamine D2 agonist in the treatment of parkinsonism. Mov. Disord..

[51] Swart PJ, De Zeeuw RA (1992). Extensive gastrointestinal metabolic conversion limits the oral bioavailability of the dopamine D2 agonist N-0923 in freely moving rats. Die Pharmazie.

[52] Pfeiffer RF (2005). A promising new technology for Parkinson's disease. Neurology.

[53] Johnston TH, Fox SH, Brotchie JM (2005). Advances in the delivery of treatments for Parkinson's disease. Expert Opin. Drug Deliv..

[54] Sieb JP (2015). Caregivers' and physicians' attitudes to rotigotine transdermal patch versus oral Parkinson's disease medication: an observational study. Curr. Med. Res. Opin..

[55] Belluzzi JD, Domino EF, May JM, Bankiewicz KS, McAfee DA (1994). N-0923, a selective dopamine D2 receptor agonist, is efficacious in rat and monkey models of Parkinson's disease. Mov. Disord..

[56] Scheller D, Ullmer C, Berkels R, Gwarek M, Lubbert H (2009). The in vitro receptor profile of rotigotine: a new agent for the treatment of Parkinson's disease. Naunyn-Schmiedeberg's Arch. Pharmacol..

[57] Elshoff JP, Braun M, Andreas JO, Middle M, Cawello W (2012). Steady-state plasma concentration profile of transdermal rotigotine: an integrated analysis of three, open-label, randomized, phase I multiple dose studies. Clin. Ther..

[58] Marrinan S, Emmanuel AV, Burn DJ (2014). Delayed gastric emptying in Parkinson's disease. Mov. Disord..

[59] Fasano A, Visanji NP, Liu LW, Lang AE, Pfeiffer RF (2015). Gastrointestinal dysfunction in Parkinson's disease. Lancet Neurol..

[60] Ray Chaudhuri K (2016). Non-oral dopaminergic therapies for Parkinson’s disease: current treatments and the future. Npj Parkinson's Dis..

[61] Isaacson S (2017). Apomorphine subcutaneous injection for the management of morning akinesia in parkinson's disease. Mov. Disord. Clin. Practice.

[62] Reuter I, Ellis CM, Ray Chaudhuri K (1999). Nocturnal subcutaneous apomorphine infusion in Parkinson's disease and restless legs syndrome. Acta Neurol. Scand..

[63] Ray Chaudhuri K (2012). Improvements in nocturnal symptoms with ropinirole prolonged release in patients with advanced Parkinson's disease. Eur. J. Neurol..

[64] Romigi A (2006). Effect of cabergoline added to levodopa treatment on sleep-wake cycle in idiopathic Parkinson's disease: an open label 24-hour polysomnographic study. J. Neural Transm..

[65] Trenkwalder C (2012). Rotigotine transdermal system for the management of motor function and sleep disturbances in Parkinson’s disease: Results from a 1-year, open-label extension of the RECOVER study. Basal Ganglia.

[66] Pagonabarraga J (2015). Transdermal Rotigotine Improves Sleep Fragmentation in Parkinson's Disease: Results of the Multicenter, Prospective SLEEP-FRAM Study. Parkinson's Dis..

[67] Calandra-Buonaura G (2016). Rotigotine objectively improves sleep in parkinson's disease: an open-label pilot study with actigraphic recording. Parkinson's Dis..

[68] Wang, Y., Yang, Y. C., Lan, D. M., Wu, H. & Zhao, Z. X. An observational clinical and video-polysomnographic study of the effects of rotigotine in sleep disorder in Parkinson's disease. *Sleep & breathing*=* Schlaf & Atmung*, doi:10.1007/s11325-016-1414-0 (2016). 10.1007/s11325-016-1414-0 27726069

[69] Pierantozzi M (2016). Rotigotine may improve sleep architecture in Parkinson's disease: a double-blind, randomized, placebo-controlled polysomnographic study. Sleep Med..

[70] Wang Y (2016). Effects of Rotigotine on REM sleep behavior disorder in Parkinson disease. J. Clin. Sleep Med..

[71] Giladi N, Fichtner A, Poewe W, Boroojerdi B (2010). Rotigotine transdermal system for control of early morning motor impairment and sleep disturbances in patients with Parkinson's disease. J. Neural Transm..

[72] Vallderiola F (2015). Effects of night-time use of rotigotine on nocturnal symptoms in Parkinson's disease. Parkinson's Dis..

[73] Ceballos-Baumann A, Hack HJ (2011). Rotigotine transdermal patch in combination therapy for Parkinson's disease--observations in routine clinical practice. Curr. Med. Res. Opin..

[74] Kassubek J (2014). Rotigotine transdermal system and evaluation of pain in patients with Parkinson's disease: a post hoc analysis of the RECOVER study. BMC Neurol..

[75] Rascol O (2016). A randomized controlled exploratory pilot study to evaluate the effect of rotigotine transdermal patch on parkinson's disease-associated chronic pain. J. Clin. Pharmacol..

[76] Canesi M, Mariani CB, Isaias IU, Pezzoli G (2010). Night-time use of rotigotine in advanced Parkinson's disease. Funct. Neurol..

[77] Kim JM (2015). Rotigotine transdermal system as add-on to oral dopamine agonist in advanced Parkinson's disease: an open-label study. BMC Neurol..

[78] Bhidayasiri R (2014). Nocturnal journey of body and mind in Parkinson’s disease: the manifestations, risk factors and their relationship to daytime symptoms. Evidence from the NIGHT-PD study. J. Neural Transm..

